# Neuregulin 4 Boosts the Efficacy of Anti-ERBB2 Neutralizing Antibodies

**DOI:** 10.3389/fonc.2022.831105

**Published:** 2022-05-18

**Authors:** Carmen Miano, Donatella Romaniello, Martina Mazzeschi, Alessandra Morselli, Silvia Da Pra, Francesca Sacchi, Chiara Bongiovanni, Michela Sgarzi, Elvira Pantano, Mattia Lauriola, Gabriele D’Uva

**Affiliations:** ^1^ National Laboratory of Molecular Biology and Stem Cell Engineering, National Institute of Biostructures and Biosystems (INBB), Bologna, Italy; ^2^ Centre for Applied Biomedical Research (CRBA), University of Bologna, Bologna, Italy; ^3^ Department of Experimental, Diagnostic and Specialty Medicine (DIMES), University of Bologna, Bologna, Italy; ^4^ Scientific and Technological Pole, IRCCS MultiMedica, Milan, Italy

**Keywords:** breast cancer, neuregulin 4 (NRG4), ERBB2 (HER2), HER2-targeted agents, neuregulin 1 (NRG1), HER2+ breast cancer, pertuzumab (Perjeta), trastuzumab (Herceptin)

## Abstract

ERBB4 is a tyrosine kinase receptor reported to exert both oncogenic and tumor suppressor activities. These paradoxical effects were suggested to stem from different ERBB4 homo-/hetero-dimers and/or isoforms. By stratifying breast cancer patients for clinical and molecular subtypes and ERBB4 mRNA abundance, we here report that higher ERBB4 levels correlate with longer relapse-free survival in breast cancer patients of HER2-enriched and luminal A molecular subtypes, proposing a cancer-protecting role for this receptor in these specific subgroups. We also observed that HER2-enriched breast cancers express intermediate ERBB4 mRNA levels compared to luminal and triple-negative/basal-like subgroups, which displayed the highest and the lowest levels, respectively. Inspired by these clinical data, we tested the activation of ERBB4 by Neuregulins as a potential anticancer strategy for HER2+ breast cancers. To this end, we employed two HER2+ breast cancer cellular models (BT474 and SKBR3), which express intermediate/high and low ERBB4 levels, respectively. Cell proliferation and motility were evaluated on these cellular models following treatments with Neuregulin 1 (NRG1), which activates both ERBB3 and ERBB4, or Neuregulin 4 (NRG4), which specifically activates ERBB4. Both NRG1 and NRG4 were used alone or in combination with anti-ERBB2 neutralizing antibodies, namely trastuzumab and pertuzumab. *In vitro* treatment with NRG1 on BT474 cells restrained cell growth and reduced the anti-proliferative efficacy of trastuzumab. In contrast, treatment with NRG1 on SKBR3 cells increased cell proliferation and migration, and partially or completely impaired the anti-proliferative/anti-migratory action of trastuzumab and/or pertuzumab. Importantly, in both the cell lines, treatment with NRG4 robustly potentiated the anti-proliferative action of trastuzumab and pertuzumab. Collectively, our data in HER2+ breast cancer cells highlight that NRG1 may exert both pro- and anti-proliferative effects, and may reduce the efficacy of anti-HER2 agents, whereas NRG4 may boost the anti-proliferative effects of anti-ERBB2 agents. We propose a provocative paradigm shift in the field of growth factors in cancer progression, suggesting the administration of ERBB4 ligands, such as Neuregulin 4, as a strategy to improve the efficacy of anti-ERBB2 agents.

## Introduction

The epidermal growth factor receptor family consists of four members, ERBB1 (also known as HER1 or EGFR), ERBB2 (HER2), ERBB3 (HER3), and ERBB4 (HER4), which are all important regulators of normal mammary gland development and physiology, and aberrations in their signaling have been linked to breast tumorigenesis [reviewed in (1–3)]. During puberty and pregnancy EGFR, ERBB2, and ERBB3 were demonstrated to drive the proliferation of mammary epithelial cells [reviewed in (4–6)]. ERBB2 is amplified in about 30% of breast tumors, and ERBB2 amplification emerged as a significant predictor of both overall survival and time to relapse in patients with breast cancer [reviewed in (1)]. Despite not being able to directly bind ligands, ERBB2 acts as the preferred hetero-dimerization partner for the other ERBB members, amplifying and diversifying the downstream signaling cascades (7) [reviewed in (1–3)]. In clinics, ERBB2 target therapies by monoclonal antibodies or tyrosine kinase inhibitors are the gold standard to treat ERBB2-overexpressing (HER2+) breast cancer patients [reviewed in (1)]. ERBB3-ERBB2 heterodimers are considered the most powerful oncogenic driver units among ERBB potential combinations (8) [reviewed in (1, 9)]. Intriguingly, higher ERBB3 expression levels correlate with lower relapse-free survival in basal-like/triple-negative (HER2-/ER-/PR-) breast cancer patients, and NRG1/ERBB3/ERBB2 axis was shown to promote anchorage-independent cell growth in basal-like/triple-negative breast cancer cells (10). EGFR (also known as ERBB1) is associated with cancer progression in several cancer types, including lung, head and neck, colorectal and pancreatic cancers [reviewed in (1–3)].

ERBB4 is the only ERBB receptor that is not necessary for the proliferation of breast epithelial cells in puberty and early pregnancy; however, it drives cell differentiation in late pregnancy and early lactation (11, 12) [reviewed in (13, 14)]. In line with a positive role in cell differentiation and/or apoptosis, increased ERBB4 expression was found associated with longer relapse-free survival (15–17), disease-free survival (17, 18), and overall survival (15, 17–20) in breast cancer patients [reviewed in (13, 21)]. Nevertheless, a few reports evidenced a correlation between increased levels of ERBB4 and an unfavorable clinical outcome, such as reduced overall survival and relapse-free survival (22, 23). Furthermore, an association between ERBB4 overexpression and chemoresistance to endocrine therapies and/or poorer prognosis has also been described (23–25) [reviewed in (13, 26, 27)]. Based on this evidence, ERBB4 has been reported to exert both oncogenic and tumor suppressor activities [reviewed in (21)]. These paradoxical effects were suggested to stem from different ERBB4 homo-/hetero-dimers and/or isoforms. Alternative splicing of ERBB4 may produce four different isoforms, which differ in the extracellular juxtamembrane domain (JMa versus JMb) and/or in the cytoplasmic domain (CYT1 versus CYT2). JMa-CYT1 isoform is predominantly expressed and considered the canonical ERBB4 transcript [reviewed in (21)]. Cleavage of JMa-CYT1 by γ-secretase determines the release of the cytoplasmic region of ERBB4 (the 4ICD) from the plasma membrane. In the mouse model, the overexpression of the canonical ERBB4 (JMa-CYT1) isoform in the breast tissue suppresses mammary terminal end bud differentiation and induces neoplastic mammary lesions (28). In contrast, breast-specific expression of the canonical CYT1 isoform of 4ICD reduces the proliferation of the ductal epithelium and induces lactogenic differentiation (29). These opposite effects triggered by the full-length ERBB4 transgene and its cytoplasmic region (4ICD-CYT1) were suggested to derive from the fact that full-length ERBB4 can heterodimerize with other ERBB family receptors, whereas the ERBB4-ICD transduces only homotypic ERBB4 signaling [reviewed in (21)]. In contrast to the 4ICD-CYT1, breast-specific expression of the CYT2 isoform of the 4ICD (4ICD-CYT2) was shown to induce epithelial hyperplasia (29).

ERBB4 can be activated by multiple ligands, including betacellulin (BTC), epiregulin (EREG), heparin-binding EGF-like growth factor (HBEGF), neuregulin 1 (NRG1), neuregulin 2 (NRG2), neuregulin 3 (NRG3), and neuregulin 4 (NRG4) [reviewed in (1–3, 30)]. In particular, EREG, HBEGF, and BTC bind to EGFR and ERBB4, NRG1 and NRG2 may activate ERBB3 and ERBB4, and NRG3 and NRG4 specifically activate ERBB4 [reviewed in (1–3, 30)]. Interestingly, the activation of ERBB4 by ligands (NRG1, HBEGF) has been shown to inhibit proliferation and/or promote differentiation in human breast normal and cancer cell lines (31, 32).

Generally, breast cancer is classified into different clinical and molecular subgroups. Clinical breast cancer subtypes are represented by HER2+, hormone-responsive (also known as “hormone receptor-positive”, “hormone responsive” or “luminal” tumors), and triple-negative (negative for the expression of estrogen receptor, progesterone receptor, and HER2) [reviewed in (33, 34)]. Molecular classification of breast cancer includes basal-like, HER2-enriched, luminal A, luminal B, and normal-like (35–37) [reviewed in (33, 34, 38)].

Our project aimed to analyze the association between ERBB4 and different breast cancer clinical and molecular subtypes, in order to unveil potential strategies to exploit the modulation of its cellular signaling as a potential anticancer strategy.

## Results

### ERBB4 Levels Correlate With Longer Survival in Breast Cancer Patients of Luminal and HER2+ Subtypes

We started our project by analyzing the association between ERBB4 expression levels and breast cancer relapse-free patients’ survival (RFS), namely the time that the patient survives without any cancer sign after primary treatment. To this end, a cohort of 4929 breast cancer patients, whose data are publicly available online, has been stratified into three groups (trichotomization) according to the expression levels of ERBB4, and the relapse-free survival probability in lower tercile versus upper tercile was evaluated during a follow-up period of 250 months after tumor resection (please refer to the “Material and methods” section for further details regarding relapse-free patients’ survival analyses). In line with the majority of previous reports (15–17) [reviewed in (13, 21)], higher expression of ERBB4 was predictive of a better relapse-free patients’ survival, with a hazard ratio (HR) of 0.53 and *p-value* < 1E-16 (**Supplementary Figure S1A**). Next, we characterized the mRNA expression of ERBB4 in publicly available datasets of breast cancer specimens and normal breast tissue (technical details are provided in the “Material and methods” section). To this aim, the analysis of a cohort of 114 normal breast tissues and 1097 breast primary tumors showed a trend toward reduced *ERBB4* mRNA expression in neoplastic specimens (**Supplementary Figure S1B**). The reduced expression of *ERBB4* did not likely rely on increased DNA methylation, since the ERBB4 promoter appeared hypomethylated both in normal and tumor samples with only a minimal increase in oncologic specimens compared to normal tissue (please consult the “Material and methods” section for further details about hypomethylation status) (**Supplementary Figure S1C**). These data demonstrate that *ERBB4* expression is modestly reduced in breast cancer tissues and that its expression is inversely correlated to cancer progression, thus suggesting that ERBB4 may act as a tumor suppressor in breast cancer patients.

**Figure 1:**
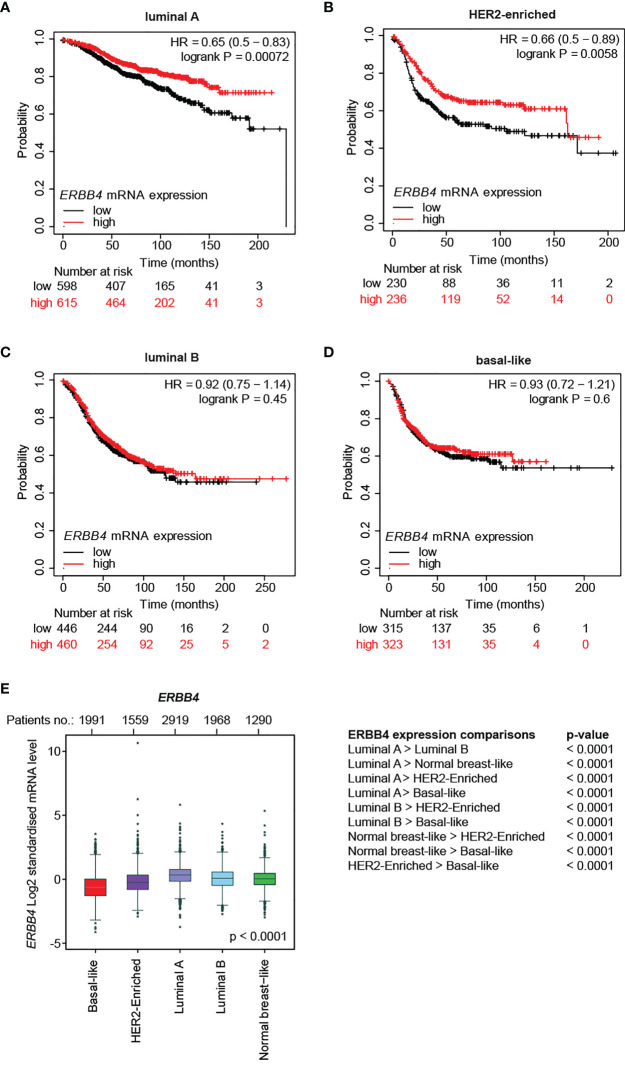
*ERBB4* mRNA levels in breast cancer patients stratified for molecular subtypes and their correlation with relapse-free patients’ survival. **(A–D)** Relapse-free survival (RFS) of breast cancer patients stratified for *ERBB4* mRNA levels (lower versus upper tercile) in molecular subtypes, namely **(A)** luminal A (n = 1809 patients), **(B)** HER2-enriched (n = 695 patients), **(C)** luminal B (n = 1353 patients), and **(D)** basal-like (n = 953 patients). Data on normal-like subgroup are not provided due to the insufficient patient number; **(E)** *ERBB4* mRNA expression levels in breast cancer patients stratified for molecular subtypes (PAM50, n = 9,727 patients); numerical data are presented as mean (error bars show s.e.m.) and * represents the expression levels of single patients falling outside the error bars.

Afterward, we evaluated the correlation between *ERBB4* mRNA abundance and relapse-free survival in different molecular subtypes. To this end, breast cancer patients belonging to specific molecular subtypes were stratified into three groups according to their expression levels of *ERBB4* mRNA. The stratification was performed by Prediction Analysis of Microarray 50 (PAM50), a 50-gene signature that classifies breast cancers into five molecular intrinsic subtypes: basal-like, HER2-enriched, luminal A, luminal B, and normal-like. RFS was calculated comparing lower tercile versus upper tercile during a follow-up period of 250 months after tumor resection. Importantly, we found that higher *ERBB4* mRNA levels predict longer relapse-free patients’ survival in luminal A and HER2-enriched subtypes (**Figures 1A, B**). In contrast, no significant association was observed between *ERBB4* mRNA levels and patients’ survival in luminal B and basal-like tumor subtypes (**Figures 1C, D**).

We then analyzed ERBB4 expression levels in breast cancer patients stratified for molecular subtypes (PAM50). Our data show that luminal A/B and normal-like breast cancer subtypes display the highest levels of ERBB4 expression, the HER2-enriched subtype shows intermediate levels, whereas the lowest levels are detected in the basal-like breast subtype (**Figure 1E**). Consistently, ERBB4 was more expressed in breast cancer cell lines of the luminal subgroup (**Supplementary Figures S2A,B**). We also analyzed the expression levels of *ERBB4* in breast cancer patients stratified for clinical subtypes. Our data show that the luminal subtype displays increased expression of *ERBB4* mRNA, whereas HER2+ and triple-negative subgroups had reduced levels (**Supplementary Figure S2C**). Similar results were obtained by the analysis of breast cancer cell lines. Indeed, their stratification for clinical subtypes showed that *ERBB4* is mainly expressed in the hormone-responsive (luminal) subtype (**Supplementary Figure S2D**). Intriguingly, the ERBB4 promoter appeared hypomethylated in normal tissues as well as in all clinical tumor subtypes, with minor variations (**Supplementary Figure S2E**). These data suggest that the low and intermediate expression levels of *ERBB4* mRNA in HER2+ and triple-negative breast cancers are not likely consequent to the increased DNA methylation.

Collectively, our data suggest a potential tumor-suppressive role for ERBB4 in luminal A and HER2+ breast cancers, which could be manipulated to reduce tumor progression.

### Treatment of BT474 (HER2+) Breast Cancer Cells With Neuregulin 1 Reduces Cell Proliferation, Despite Impairing the Efficacy of Trastuzumab

**Figure 2:**
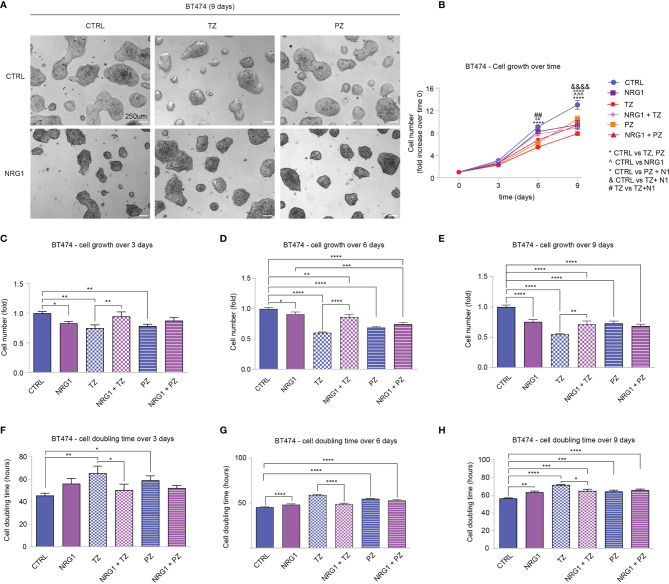
Treatment with NRG1 in BT474 (HER2+) breast cancer cells restrains cell proliferation, despite impairing trastuzumab efficacy. **(A–H)** Cell proliferation analysis over time. BT474 cells were cultured *in vitro* and treated with/without NRG1 (10 ng/mL), alone or in combination with trastuzumab (10 µg/mL) or pertuzumab (10 µg/mL) every 3 days and analyzed up to 9 days. Representative images at 9 days post-treatment are provided in **(A)** scale bar 250µm; Cell growth over time, normalized to the seeded cells at 0 days, is provided in **(B)**; cell growth and cell doubling time at 3 days, 6 days, and 9 days are provided in **(C–H)** respectively. In all panels, numerical data are presented as mean (error bars show s.e.m.); statistical significance was determined using two-way ANOVA in **(B)** and one-way ANOVA in **(C–H)** followed by Tukey’s test; (*) p < 0.05, (**, °°, ##) p < 0.01, (***, ^^^) p < 0.001, (****, °°°°, &&&&) p < 0.0001.

Clinical data supported a link between ERBB4 and increased survival in luminal and HER2+ breast cancer patients. Of note, ERBB4 activation, induced by stimulation with neuregulin 1 (NRG1), has been shown to promote the differentiation and impair the growth of SUM44 breast cancer cells, which belong to the luminal subtype (32) and express very high levels of ERBB4 (**Supplementary Figure S2B**) and low levels of ERBB2 (10). Thus, we decided to test whether the activation of ERBB4 signaling by neuregulins may restrain the aggressiveness of HER2+ breast cancers, if ERBB2 is inhibited by anti-HER2 agents. To test this hypothesis, we first employed BT474 cells, a human breast cancer ERBB2-overexpressing cell line, which expresses ERBB4 at intermediate/high levels (**Supplementary Figure S2B**). To neutralize ERBB2 we employed two anti-ERBB2 humanized monoclonal antibodies, namely trastuzumab and pertuzumab, currently used in clinics to treat HER2+ breast cancer patients. In particular, trastuzumab binds to the extracellular domain IV of HER2, strongly inhibiting its ligand-independent activation as homodimer, which mainly occurs when ERBB2 is overexpressed (1, 39–41). However, trastuzumab is less effective in the presence of a ligand (42). Pertuzumab binds to the extracellular domain II of HER2, essential for dimerization, thus inhibiting ligand-dependent HER2 hetero-dimerization (41). BT474 cells were therefore treated with neuregulin 1 (NRG1) and anti-ERBB2 agents (pertuzumab or trastuzumab), alone or in combination, and their proliferation was monitored by cell counting every 3 days for a total of 9 days. As expected, treatment with anti-HER2 agents trastuzumab and pertuzumab reduced cell number (**Figures 2A–E**), increasing cell doubling time (**Figures 2F–H**). Interestingly, treatment with NRG1 reduced cell growth (**Figures 2A–E**), thus increasing cell doubling time (**Figures 2F–H**). Stimulation with NRG1 did not have an impact on the anti-proliferative efficacy of pertuzumab; however, it impaired the anti-proliferative activity of trastuzumab (**Figures 2A–E**), thus decreasing cell doubling time (**Figures 2F–H**). Overall, these data suggest that NRG1 reduces the proliferation of BT474 (HER2+) cells, despite impairing the efficacy of trastuzumab.

### Treatment Of SKBR3 (HER2+) Breast Cancer Cells with Neuregulin 1 Increases Cell Proliferation and Migration, and Completely or Partially Impairs the Efficacy of Anti-ERBB2 Targeting Drugs Trastuzumab and Pertuzumab

**Figure 3:**
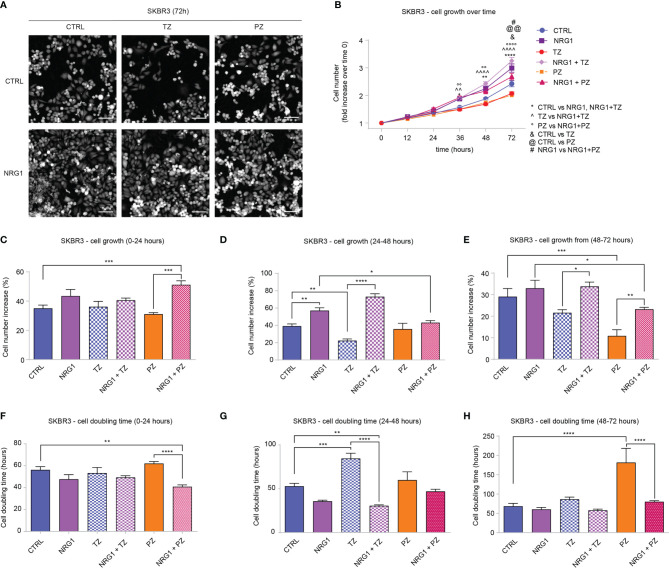
Treatment with NRG1 in SKBR3 (HER2+) breast cancer cells induces cell proliferation and impairs trastuzumab and pertuzumab efficacy. **(A–H)** Time-lapse analysis over time by Livecyte technology. SKBR3 cells were cultured *in vitro* and treated with/without NRG1 (10 ng/mL), alone or in combination with trastuzumab (10 µg/mL) or pertuzumab (10 µg/mL) and monitored up to 72 hours. Representative images at 72 hours post-treatment are provided in **(A)** scale bar 75 µm; Cell growth over time, normalized to cell number at 0 hours, is provided in **(B)**; Cell growth and cell doubling time during the first day (0-24 hours), second day (24-48 hours), and third day (48-72 hours) are provided in **(C–H)** respectively. In all panels, numerical data are presented as mean (error bars show s.e.m.); statistical significance was determined using two-way ANOVA in **(B)** and one-way ANOVA in **(C–H)** followed by Tukey’s test; (*, &, #) p < 0.05, (**, ^^, °°, @@) p < 0.01, (***) p < 0.001, (****, ^^^^, °°°°) p < 0.0001.

To strengthen our observations, we evaluated the impact of NRG1 on a second HER2+ cellular model, SKBR3 cells, which are characterized by lower expression levels of ERBB4 compared to BT474 cells (**Supplementary Figure S2B**). SKBR3 cells were treated with NRG1 and the effects were monitored by time-lapse imaging over time up to 3 days with Livecyte technology. As expected, treatment with anti-HER2 agents trastuzumab and pertuzumab reduced cell number (**Figures 3A, B** and **Supplementary Figure S3A**) and increased cell doubling time (**Supplementary Figure S3B**). In contrast to BT474 cells, treatment with NRG1 significantly increased SKBR3 cell number (**Figures 3A, B** and **Supplementary Figure S3A**), reducing cell doubling time (**Supplementary Figure S3B**). NRG1 also completely inhibited trastuzumab efficacy and reduced pertuzumab action (**Figures 3A, B** and **Supplementary Figures S3A, B**). To better evaluate the impact of single or combinatorial treatment with NRG1 and anti-HER2 agents we analyzed the dynamics of cell growth (**Figures 3C–E**) and cell doubling time (**Figures 3F–H**) over time. Our data show that NRG1-induced proliferation peaks during the second day after the stimulation (**Figures 3D, G**), although a trend towards an increase was also observed during the first day (**Figures 3C, F**) and the third day (**Figures 3E, H**). The antiproliferative efficacy of trastuzumab was observed after 24 hours; however, it was completely impaired by NRG1 co-treatment (**Figures 3D, G**). The antiproliferative action of pertuzumab was evident after 48 hours, and it was partially reduced by NRG1 co-treatment (**Figures 3E, H**).

Afterward, we evaluated the impact of NRG1 and anti-HER2 agents on cell cycle stages. To this end, SKBR3 cells were treated for 48 hours with NRG1 and anti-HER2 agents (trastuzumab and pertuzumab), alone or in combination, and their cell cycle was evaluated by Propidium Iodide staining and flow-cytometry analysis. In line with a proliferative role, NRG1 induced a modest increase in S-phase, coupled with a mild reduction in G0/G1 phase (**Supplementary Figure S4**). These NRG1-induced effects were evident also after trastuzumab co-treatment (**Supplementary Figure S4**). On the other hand, pertuzumab increased the percentage of cells in G0/G1 phase, reducing those in S and G2/M phases, and these effects were reduced by co-treatment with NRG1 (**Supplementary Figure S4**).

Overall, our data suggest that NRG1 induces the proliferation of SKBR3 (HER2+) breast cancer cells, and partially or completely impairs the efficacy of anti-ERBB2 agents.

**Figure 4:**
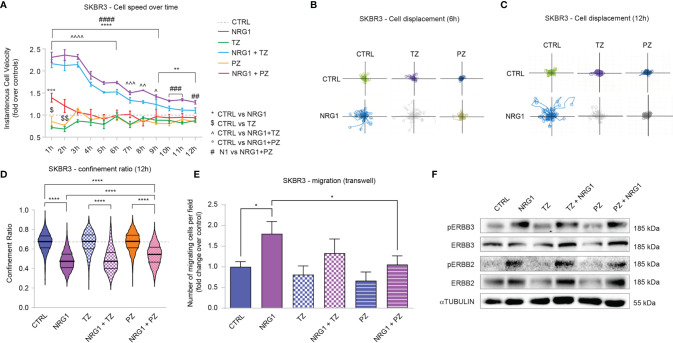
Treatment with NRG1 in SKBR3 (HER2+) breast cancer cells induces cell motility and reduces trastuzumab and pertuzumab efficacy. **(A)** Cell speed analysis over time by Livecyte technology of SKBR3 cells treated with/without NRG1 (10 ng/mL), alone or in combination with trastuzumab (10 µg/mL) or pertuzumab (10 µg/mL). Cell velocity has been detected every hour up to 12 hours and normalized to control cells (dotted line); **(B, C)** SKBR3 cell displacement analysis by Livecyte technology. Representative track plots of SKBR3 treated with/without NRG1 (10 ng/mL), alone or in combination with trastuzumab (10 µg/mL) or pertuzumab (10 µg/mL), up to 6 and 12 hours are provided in **(B, C)**, respectively; **(D)** Confinement ratio analysis of SKBR3 cells by Livecyte technology. Violin plots of SKBR3 treated with/without NRG1 (10ng/mL), alone or in combination with trastuzumab (10 µg/mL) or pertuzumab (10 µg/mL) for 12 hours; **(E)** Transwell migration assay performed on SKBR3 cells treated with/without NRG1 (10 ng/mL), alone or in combination with trastuzumab (10 µg/mL) or pertuzumab (10 µg/mL) for 20 hours; **(F)** Western blot analysis of phosphorylated (active) and total ERBB3 and ERBB2 protein levels in lysates of SKBR3 cells cultured *in vitro* and treated with/without NRG1 (10 ng/mL), alone or in combination with trastuzumab (10 µg/mL) or pertuzumab (10 µg/mL) for 30 minutes. In all panels, numerical data are presented as mean (error bars show s.e.m.); statistical significance was determined using two-way ANOVA in **(A)** and one-way ANOVA in **(D, E)** followed by Tukey’s test; (*, $, ^) p < 0.05, (**, $$, ^^) p < 0.01, (***, ^^^, °°°, ###) p < 0.001, (****, ^^^^, ####) p < 0.0001.

Cell motility is a key mechanism involved in tumor dissemination and metastatic spread. We thus analyzed SKBR3 cell migration in response to combinatorial treatments of NRG1, trastuzumab, and pertuzumab. Stimulation with NRG1 fosters SKBR3 cell migration as evidenced by a robust increase in cell velocity one hour after the treatment, which then remains significantly augmented although progressively reducing during the following twelve hours (**Figure 4A**). The degree of directional versus random migration was estimated by calculating the cell displacement, namely the position of cells and their trajectories over time relative to their point of origin, and cell confinement ratio, namely the ratio of the length of the direct path between the initial and the current position over its current track length. In this regard, treatment with NRG1 increased cell displacement (**Figures 4B, C**) and reduced the confinement ratio (**Figure 4D**). These data suggest that NRG1 induces the directional migration of SKBR3 cells. Within the first hours of treatment, trastuzumab alone slightly reduced cell migration, whereas pertuzumab showed a trend toward a reduction (**Figure 4A**). Trastuzumab was unable to significantly reduce the NRG1-induced cell motility, in terms of cell velocity (**Figure 4A**), cell displacement (**Figures 4B, C**), and cell confinement ratio (**Figure 4D**). In contrast, the treatment with pertuzumab was very efficient in preventing cell motility induced by NRG1 (**Figure 4A**), as well as directional migration as evidenced by the suppression of the massive cell displacement at 6 and 12 hours post-treatment (**Figures 4B, C**) and the partial inhibition of the reduction in cell confinement ratio (**Figure 4D**). The impact of NRG1 on SKBR3 cell motility was also evaluated by transwell migration assay, confirming the ability of NRG1 in fostering cell migration, which could be significantly inhibited by pertuzumab (**Figure 4E**).

We also analyzed potential differences in morphological parameters of SKBR3 cells upon the combinatorial treatment with NRG1, trastuzumab, and pertuzumab. Consistent with the positive role in cell migration and proliferation, NRG1 induced an increase in cell area along with an increment in length-to-width ratio, which was partially prevented by the treatment with pertuzumab or trastuzumab (**Supplementary Figures S5A, B**).

Overall, these data show that the treatment of SKBR3 cells with NRG1 supports cell proliferation and migration, and partially or completely impairs the anti-proliferative/anti-migratory action of trastuzumab and/or pertuzumab. Of note, NRG1 is a well-known activator of ERBB3/ERBB2 dimers, which are known to play a role in the context of HER2+ breast cancer aggressiveness. In line with this reasoning, the stimulation with NRG1 robustly induces the activation of ERBB3 and ERBB2 in SKBR3 cells, as evidenced by the increased phosphorylation status (**Figure 4F**). Notably, treatment with trastuzumab decreased total protein levels of ERBB2 and to a lesser extent ERBB3 (**Figure 4F**), consistent with receptor degradation as previously described (43, 44) [reviewed in (45, 46)]. In our hands, also pertuzumab reduced total levels of ERBB2 and, in line with previous studies, ERBB3 (47) (**Figure 4F**). However, trastuzumab and to a lesser degree pertuzumab were not able to efficiently prevent ERBB2 activation (**Figure 4F**). Thus, the observed effects induced by NRG1 on SKBR3 cells are likely mediated by ERBB3/ERBB2 heterodimers and could be only partially inhibited by co-treatment with ERBB2 targeting agents.

### Treatment of HER2+ Cancer Cells With Neuregulin 4 Boosts the Anti-Proliferative Effects of Anti-ERBB2 Agents

**Figure 5:**
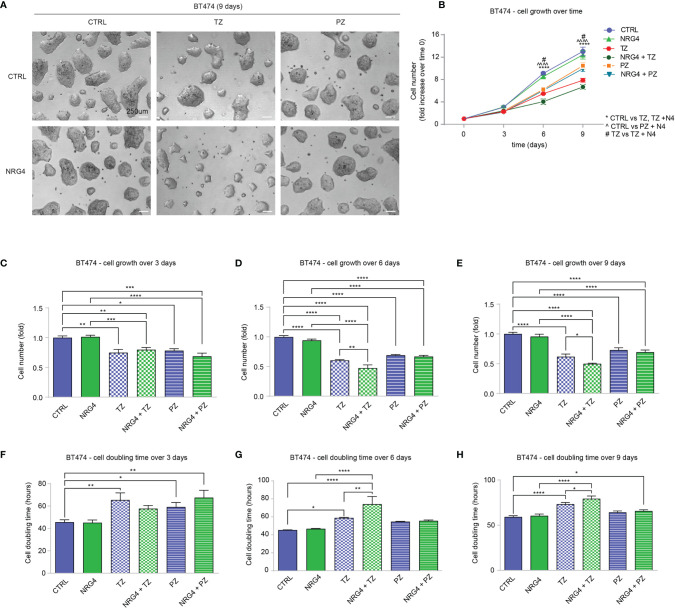
Treatment with NRG4 in BT474 (HER2+) breast cancer cells boosts trastuzumab efficacy. **(A–H)** Cell proliferation analysis over time. BT474 cells were cultured *in vitro* and treated with/without NRG4 (10 ng/mL), alone or in combination with trastuzumab (10 µg/mL) or pertuzumab (10 µg/mL) every 3 days and analyzed up to 9 days. Representative images at 9 days post-treatment are provided in **(A)** scale bar 250µm. Cell growth over time, normalized to the seeded cells at 0 days, is provided in **(B)**; cell growth and cell doubling time at 3 days, 6 days, and 9 days are provided in **(C–H)** respectively. In all panels, numerical data are presented as mean (error bars show s.e.m.); statistical significance was determined using two-way ANOVA in **(B)** and one-way ANOVA in **(C–H)**, followed by Tukey’s test; (*) p < 0.05, (**) p < 0.01, (***) p < 0.001, (****, ^^^^) p < 0.0001.

Our data suggested that the treatment of HER2+ breast cancer cells with NRG1 may sustain or restrain cell proliferation, as well as reduce the efficacy of ERBB2 targeting agents. The pro-proliferative effect of NRG1 likely depends on the direct activation of ERBB3/ERBB2 heterodimers. To evaluate the impact of the activation of ERBB4 on proliferation and motility of HER2+ breast cancer cells, avoiding the undesired activation of ERBB3, we thus employed neuregulin 4 (NRG4), which specifically binds only ERBB4 (48) [reviewed in (1–3, 30)]. BT474 cells were therefore treated with NRG4 and anti-ERBB2 agents pertuzumab or trastuzumab, alone or in combination, and their proliferation and cell doubling time were monitored and calculated by counting the cell number every 3 days for a total of 9 days (**Figures 5A–H**). Treatment with NRG4 did not induce significant effects on cell growth and cell doubling time over 9 days experiment (**Figures 5A–H**). Importantly, NRG4 boosted trastuzumab efficacy, reducing cell number at 6 and 9 days post-treatment (**Figures 5D, E**) and increasing cell doubling time (**Figures 5G, H**). In contrast, the addition of NRG4 to pertuzumab did not enhance the anti-proliferative activity at any of the analyzed time points (**Figures 5C–H**). Overall, our data suggest that the treatment with NRG4 alone does not induce significant effects on BT474 cell proliferation; however, it boosts the efficacy of anti-ERBB2 agents, restraining BT474 cell growth when combined with trastuzumab.

**Figure 6:**
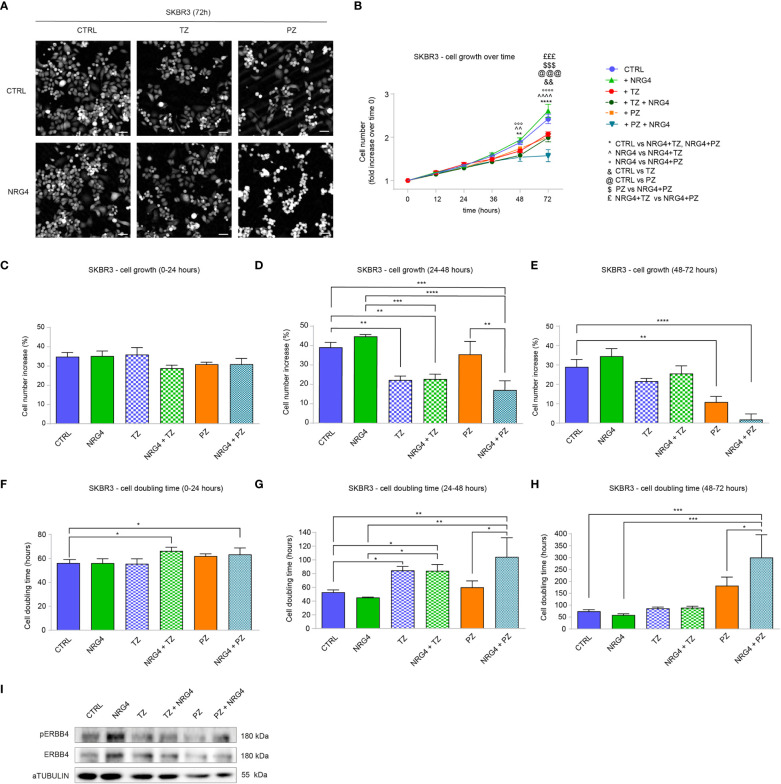
Administration of NRG4 boosts the anti-cancer efficacy of anti-HER2 agents in SKBR3 HER2+ breast cancer cells. **(A–H)** Time-lapse analysis over time by Livecyte technology. SKBR3 cells were cultured *in vitro* and treated with/without NRG4 (10 ng/mL), alone or in combination with trastuzumab (10 µg/mL) or pertuzumab (10 µg/mL) and monitored up to 72 hours. Representative images are provided at 72 hours post-treatment; scale bar 75µm; Cell growth over time, normalized to cell number at 0 hours, is provided in **(B)**; cell growth and cell doubling time during the first day (0-24 hours), second day (24-48 hours), and third day (48-72 hours) are provided in **(C–H)** respectively; **(I)** Western blot analysis of phosphorylated (active) and total ERBB4 protein levels in lysates of SKBR3 cells cultured *in vitro* and treated with/without NRG4 (10 ng/mL), alone or in combination with trastuzumab (10 µg/mL) or pertuzumab (10 µg/mL) for 30 minutes. In all panels, numerical data are presented as mean (error bars show s.e.m.); statistical significance was determined using two-way ANOVA in **(B)** and one-way ANOVA in **(C–H)**, followed by Tukey’s test; (*) p < 0.05, (**, ^^, &&) p < 0.01, (***, °°°, $$$, £££, @@@) p < 0.001, (****, ^^^^, °°°°) p < 0.0001.

Next, we evaluated the impact of NRG4 on SKBR3 cells. Upon administration of NRG4 to SKBR3 cells, we monitored the effects by time-lapse imaging over time up to 3 days (**Figures 6A, B**). NRG4 treatment on SKBR3 cells only slightly induces significant effects on cell growth and cell doubling time over 3 days (**Supplementary Figures S6A, B** and **Figures 6A, B**). However, treatment with NRG4 potentiated the anti-proliferative action of pertuzumab (**Supplementary Figures S6A, B** and **Figures 6A, B**). This additive effect was not observed by co-treatment with NRG4 and trastuzumab (**Supplementary Figures S6A, B** and **Figures 6A, B**). Importantly, by stratifying cell growth over days, we could appreciate that the synergism of NRG4 and pertuzumab was very robust 3 days after the stimulus, showing an impressive 98% reduction in cell number (**Figures 6C–E**) along with an impressive increase in cell doubling time (**Figures 6F–H**). However, the analysis of the cell cycle 48 hours after the combinatorial treatment with NRG4 and pertuzumab shows only a slight reduction in S phase compared to pertuzumab alone (**Supplementary Figure S7**), suggesting that other mechanisms, for example, the induction of cell death, may be responsible for this dramatic effect.

Regarding cell motility, we observed that NRG4 promotes a non-significant and modest reduction in cell speed by Livecyte imaging analyses, without significant synergism with pertuzumab or trastuzumab (**Supplementary Figure S8A**). In line, no important changes in cell displacement and confinement ratio were observed after treatments with NRG4 (**Supplementary Figures S8B, C**). Accordingly, the treatment with NRG4 or anti-ERBB2 agents (trastuzumab or pertuzumab) reduced cell migration in a transwell assay, despite reaching statistical significance only with pertuzumab treatment (**Supplementary Figure S8D**). In line with a suppressive role in cell proliferation and migration, the combinatorial treatment of NRG4 and pertuzumab decreased cell area and length-to-width ratio (**Supplementary Figures S9A, B**). Finally, the activation of ERBB4 after treatment with NRG4 was confirmed by the evaluation of the phosphorylated (active) ERBB4 protein levels (**Figure 6I**). Indeed, treatment with pertuzumab reduced the basal levels of ERBB4 activation, which were modestly increased by co-treatment with NRG4 (**Figure 6I**). Collectively, these data in HER2+ breast cancer cells demonstrate that NRG4 activates ERBB4 and boosts the anti-proliferative effects of anti-ERBB2 agents.

**Figure 7:**
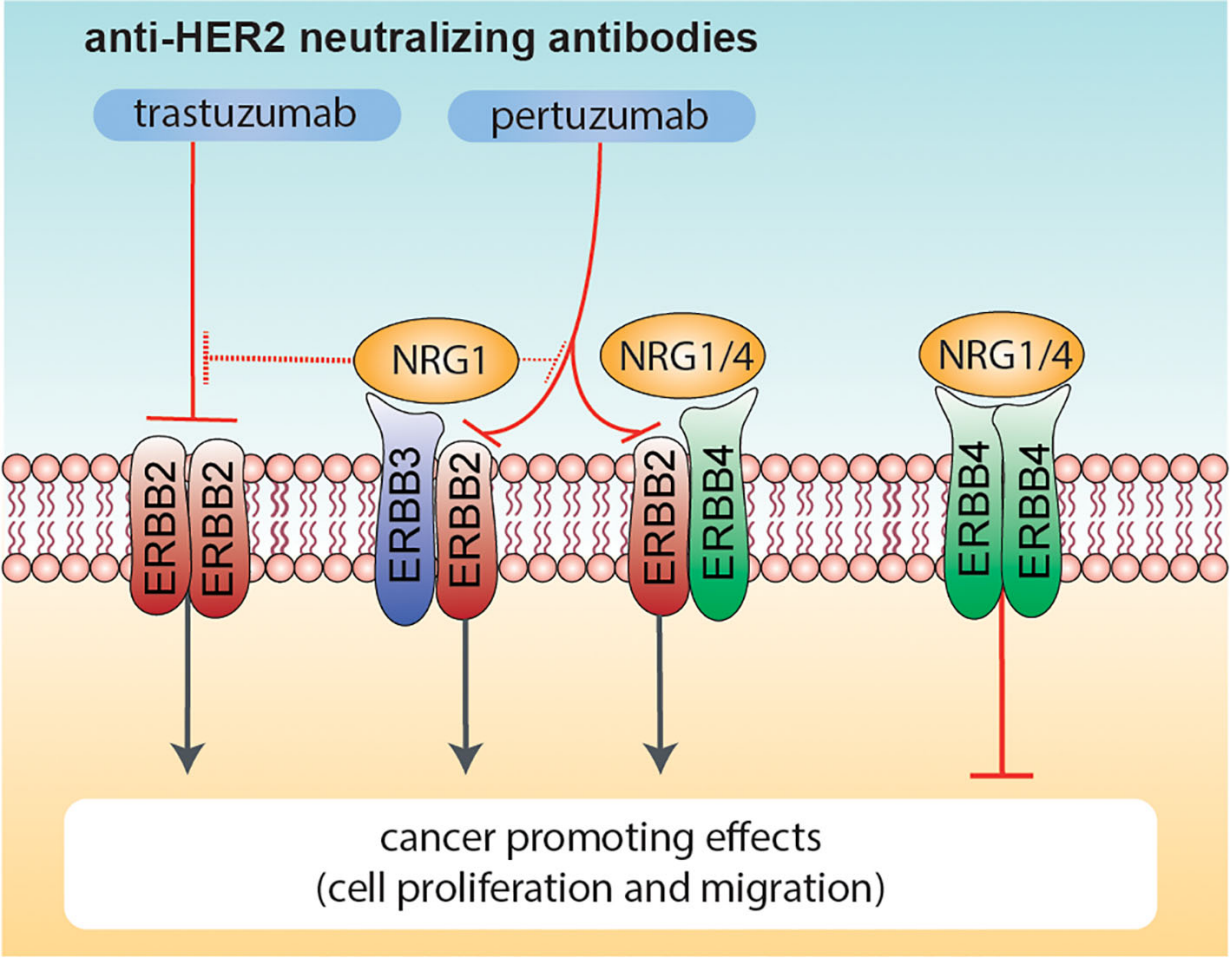
Effects of combinatorial treatments with Neuregulins and anti-ERBB2 agents on breast cancer cell proliferation. Schematic diagram summarizing the effects induced by co-administration of anti-ERBB2 agents (trastuzumab and pertuzumab) and Neuregulins (NRG1 and NRG4) on breast cancer cell proliferation. NRG1 binds to ERBB3 and ERBB4, whereas NRG4 specifically binds to ERBB4. Neuregulin-activated ERBB3 and ERBB4 preferentially heterodimerize with ERBB2 (if available), in turn promoting cancer cell proliferation. Trastuzumab robustly inhibits ERBB2 ligand-independent activation as homo-dimer, whereas pertuzumab is more efficient in inhibiting HER2 hetero-dimerization with other ERBB receptors (including ERBB3 and/or ERBB4 activated by NRG1 or NRG4). NRG1 also reduces the efficacy of anti-HER2 agents, in particular trastuzumab. In contrast, the co-treatment of NRG4 and anti-HER2 agents promotes ERBB4 homodimers activation, in turn reducing breast cancer cell proliferation.

Altogether, our data highlight that NRG1 may exert both pro- and anti-proliferative effects on HER2+ breast cancer cellular models, and may reduce the efficacy of anti-HER2 agents, whereas NRG4 consistently boosts the anti-proliferative effects of anti-HER2 agents (**Figure 7**). We thus suggest the administration of neuregulin 4 as a strategy to improve the efficacy of anti-ERBB2 neutralizing antibodies in breast cancer patients.

## Discussion

The role of ERBB4 in cancer progression, including breast cancer, is currently controversial since both tumor suppressive and oncogenic activities for ERBB4 have been documented [reviewed in (13, 21, 49)].

In line with the majority of previous clinical reports (15–17) [reviewed in (13, 21)], our clinical metanalyses support an association between higher ERBB4 levels and longer relapse-free survival in breast cancer patients. Consistent with previous observations in other tumor types [reviewed in (13)] and in support of a cancer suppressive role of ERBB4 in breast cancer, we observed a trend towards reduced *ERBB4* mRNA expression in neoplastic specimens compared to normal breast tissues.

Importantly, our analyses show that higher ERBB4 levels correlate with longer relapse-free survival only in luminal A and HER2+ subtypes, suggesting that the potential tumor-suppressive role of ERBB4 is restricted to these subtypes. Indeed, we did not appreciate significant differences in relapse-free survival of basal-like and luminal B breast cancer patients stratified for ERBB4 expression levels. The strength of our analysis relies on the evaluation of a large number of cancer samples, selection of optimal probes, exclusion of outlier arrays and stratification of patients by trichotomization. Our observation of a positive correlation between ERBB4 mRNA levels and increased relapse-free patients’ survival in the luminal A subtype is in line with a previous study showing that the activation of ERBB4 signaling by NRG1 restrains the growth of breast cancer luminal cells (32). Importantly, the luminal breast cancer subtype expresses high levels of ERBB4 (please consult **Figure 1E**) and ERBB3 (10) and quite low ERBB2 and EGFR levels (50) [reviewed in (51)]; by consequence, the activation of ERBB4 by ligands in this breast tumor subtype is expected to preferentially activate ERBB4-ERBB4 homodimers or ERBB4-ERBB3 heterodimers. Apart from a modest reduction in ERBB4 (please consult **Figure 1E**), the abundance of ERBB receptors in the luminal B subgroup does not differ that much as compared to luminal A (10). Why patients’ stratification based on ERBB4 abundance showed no difference in survival of breast cancer patients of the luminal B subtype deserves further investigation. To date, a potential protective or promoting role for ERBB4 in basal and/or triple-negative breast cancers is a debated topic, and our data do not support this hypothesis. However, previous studies on this topic were controversial. Indeed, a previous study reported that elevated ERBB4 levels correlate with increased relapse-free survival in all breast cancer clinical subtypes, including triple-negative breast cancer (16). On the other side, ERBB4 overexpression has been found associated with a poorer prognosis in breast cancers of the triple-negative subtype (24) [reviewed in (21)]. Triple-negative and basal-like breast cancer subtypes, which exhibit a high degree of gene expression profile overlap (50, 52, 53) [reviewed in (54–56)], are characterized by elevated expression of EGFR (50) [reviewed in (51)]; thus, it has been hypothesized that ERBB4-EGFR heterodimers in these breast cancer subtypes may function as oncoproteins [reviewed in (21)].

Based on the positive association between higher ERBB4 levels and longer relapse-free survival of HER2+ breast cancer patients, we performed additional analyses for the evaluation of the role of ERBB4 in HER2+ breast cancers, which to date is still unclear. Intriguingly, treatment with NRG1 on two HER2+ breast cancer cellular models, namely BT474 and SKBR3 cells, unveiled an opposite response on cell proliferation. Indeed, while NRG1 reduced the proliferation of BT474 cells, the same treatment on SKBR3 cells induced a proliferative response. However, the pro-proliferative effect of NRG1 on SKBR3 is likely due to the activation of ERBB3/ERBB2 heterodimers, since these receptors were activated by NRG1 treatment, and the co-treatment with anti-ERBB2 agents was able to partially prevent these effects. Importantly, in both BT474 and SKBR3 cells, the efficacy of anti-HER2 agents, in particular trastuzumab but also pertuzumab, was completely abolished or reduced by treatment with NRG1. These data are in line with the reported ability of NRG1 in inducing primary resistance to trastuzumab in HER2+ breast cancer cells (57). Overall, NRG1 appears to induce undesired effects on HER2+ breast cancer cells, ranging from increased proliferation and motility, and resistance to anti-ERBB2 agents.

Our data suggest that the activation of ERBB4 by NRG4 does not have a significant impact on HER2+ breast cancer cell proliferation. Importantly, treatment with NRG4 and simultaneous blockage of ERBB2, which is expected to specifically trigger the activation of ERBB4-ERBB4 homodimers, restrained the growth of HER2+ breast cancer cells more efficiently than anti-HER2 drugs alone. Thus, our study suggests the provocative administration of a growth factor as an anti-cancer strategy in cancer patients. Obviously, the interception of potential deleterious effects activated by growth factors would be critical. In this regard we here suggest the concomitant inhibition of the ERBB2 co-receptor, which can induce pro-proliferative and pro-migratory effects. However, further studies addressing how the activation of ERBB4 homodimers is responsible for the reduction in cell growth of HER2+ cells are needed. A potential mechanism may involve cell differentiation, which has been reported to be induced by ERBB4 activation (32, 58).

Of note, a common side effect of chemotherapy and targeted therapies is cardio-toxicity, which strongly impacts the quality of life and overall survival of cancer patients, regardless of the oncological prognosis [reviewed in (59–64)]. Importantly, NRG1 has been shown to protect cardiac myocytes from anthracycline-induced apoptosis (65–67). However, despite its efficacy, NRG1 is not clinically relevant as a therapeutic for cardiomyopathy induced by anti-cancer therapies because of its well-established role in preneoplastic signaling. An engineered bivalent NRG1, which preferentially induces ERBB4 homodimer formation and protects against doxorubicin-induced cardiotoxicity with reduced preneoplastic potential, has also been created (68). Here, instead, we suggest using NRG4 coupled with anti-ERBB2 agents as a promising strategy to induce the activation of ERBB4 homodimers. Further studies are needed to understand whether this combination may protect the heart from the cardiotoxic effects induced by the administration of anthracyclines and/or anti-HER2 agents.

In conclusion, here we propose a provocative therapy based on the combinatorial administration of a growth factor, namely NRG4, in combination with anti-ERBB2 antibodies as a novel anti-cancer strategy based on specific activation of ERBB4-ERBB4 homodimers.

## Material and Methods

### Bioinformatic Analysis of Breast Cancer Patients’ Data

Evaluation of mRNA expression of genes of interest in breast cancers stratified for molecular subtypes was conducted by “bc-GenExMiner” (69) (http://bcgenex.ico.unicancer.fr/). Evaluation of mRNA expression or promoter methylation of genes of interest in normal breast tissues and breast cancer specimens stratified for clinical subtypes was conducted by “UALCAN” online tool (70) (http://ualcan.path.uab.edu/). For promoter methylation analysis beta value indicates the level of DNA methylation ranging from 0 (unmethylated) to 1 (fully methylated). Different beta value cut-off has been considered to indicate hypermethylation [beta value: 0.7 - 0.5] or hypo-methylation [beta-value: 0.3 - 0.25] (71, 72).

Evaluation of mRNA expression of the gene of interest in the different breast cancer cell lines stratified for molecular and clinical subtypes was conducted by “Gene expression-based Outcome for Breast cancer Online” (GOBO) online tool (73) (http://co.bmc.lu.se/gobo/gsa.pl). GOBO gene set expression analysis in breast cancer cell lines (GSA-Cell line) includes mRNA expression data across 51 breast cancer cell lines (74).

Analyses of relapse-free survival of breast cancer patients were conducted by KM plotter online database (75) (http://kmplot.com/). In detail, the KM plotter was utilized to evaluate the correlation between ERBB4 mRNA expression levels and RFS of breast cancer patients stratified in molecular subtypes. KM plotter sources for the databases include GEO, EGA, and TCGA. Patients belonging to specific breast cancer molecular subtypes were stratified into three groups (trichotomization) according to their expression levels of *ERBB4* mRNA, and RFS after tumor resection was calculated by the Kaplan–Meier curve and log-rank test during a follow-up period of 250 months comparing lower tercile versus upper tercile. The results were shown in the Kaplan–Meier survival plots. P-value and Hazard ratio (HR) are provided. P-value < 0.05 was regarded as statistically significant by using Log-rank test. Hazard Ratios were used to estimate the effect for time-to-event end points, such as relapse-free survival. A Hazard Ratio of 1 means lack of association, a Hazard Ratio greater than 1 suggests an increased risk, and a Hazard Ratio below 1 suggests a smaller risk.

### Cell Cultures

*In vitro* experiments have been conducted in breast cell lines BT474 and SKBR3. Both BT474 and SKBR3 cells were cultured in RPMI-1640 supplemented with 10% of Fetal Bovine Serum (FBS), 1% penicillin/streptomycin, 1% L- glutamine. The cells have been grown in 10 cm plastic Petri dishes and incubated at 37°C in a humidified atmosphere of 5% CO2/air.

### Proliferation and Random/Directional Migration Analysis in Monolayer Conditions

50.000 BT474 cells/well were seeded into a six-well plate in full medium. Treatments with neuregulin 1 (10 ng/mL), neuregulin 4 (10 ng/mL), alone and in combination with trastuzumab (10 µg/mL), and pertuzumab (10 µg/mL) were added the day after seeding and repeated every 3 days. Trastuzumab and pertuzumab were added at least 30 minutes before adding NRG1 or NRG4. After respectively 3, 6, and 9 days of treatments, cells were trypsinized and manually counted using the Neubauer Chamber. Representative pictures were acquired by using EVOS™ M5000 Imaging System at 4× magnification. Then, proliferation and migration analyses on SKBR3 cells were performed using a Livecyte TM technology (Phase Focus, Sheffield, UK). 2.000 SKBR3 cells/well were seeded into a 96-well plate in full medium. The day after, cells were treated with NRG1 (10 ng/mL), NRG4 (10 ng/mL), trastuzumab (10 µg/mL), and pertuzumab (10 µg/mL), alone and in combination, before the start of the experiment. Trastuzumab and pertuzumab were added at least 30 minutes before adding NRG1 or NRG4. Images were acquired every 60 min for 72h with a 10x objective, at 37°C and 5% CO2. Data were analyzed using Cell Analysis Toolbox software (Phase Focus, Sheffield, UK). Cell proliferation was determined by the software considering the number of cells in each frame. Cell motility was evaluated by measuring cell velocity, calculated as the change in position in each frame. The degree of directional versus random migration was estimated by calculating cell displacement and cell confinement ratio. Indeed, these two parameters represent the distance a cell migrates relative to its point of origin and also consider the degree to which a cell meanders from its starting and ending points. In particular, cell displacement shows the position of cells and their trajectories over time, relative to their point of origin. Confinement ratio is the ratio of the length of the direct path between the initial and the final position over the total track length. To circumvent the problem of dependency on the cell track duration the confinement ratio was multiplied by the square root of time. Morphological and morphometrical analyses were performed by the software, calculating the area of cells in each frame, the sphericity, measuring how close to a sphere is a cell in each frame, and the length to width ratio, deriving from the calculation of how round versus elongated is a cell in each frame. For both the cell lines cell doubling time was calculated according to the following formula: experiment duration * ln(2)/ln(final concentration/initial concentration).

### Transwell Migration Assay

100.000 SKBR3 cells were seeded on the transwell inserts pre-incubated with full medium with/without neuregulin 1 (10 ng/mL), neuregulin 4 (10 ng/mL), trastuzumab (10 µg/mL), and pertuzumab (10 µg/mL), alone and in combination, for at least 1 hour. Inserts were washed in PBS and fixed with 4% paraformaldehyde, after a migration time of about 24 hours. After fixing, inserts were washed in PBS and stained with 0.5% crystal violet (Sigma-Aldrich, Saint Louis, MO, USA) for 15 minutes, as previously described (76). Inserts were rinsed in water and cells that did not cross the insert membrane were removed with a cotton swab. Pictures of cells on the bottom of the insert membrane were acquired with Leica MZ FLIII stereomicroscope and quantified with Image J software.

### Western Blot Analysis

750.000 SKBR3 cells were seeded on 6 cm plastic Petri dishes and cultured as a monolayer. The day after seeding and overnight starvation, treatments with neuregulin-1 (10 ng/mL), neuregulin-4 (10 ng/mL), trastuzumab (10 µg/mL), and pertuzumab (10 µg/mL), alone and in combination, were added.

After 30 minutes of treatments, cells were washed and scrapped in cold RIPA buffer supplemented with a protease inhibitor cocktail (P8340, Sigma-Aldrich, Saint Louis, MO, USA, 1:100) and Na_3_VO_4_ (1 mM). Protein extracts were then analyzed by western blotting, as previously described (10). Briefly, protein lysates were resolved by sodium dodecyl sulfate (SDS)-polyacrylamide gel electrophoresis and transferred to a nitrocellulose membrane (AmershamTM ProtranTM Premium 0.45 μm 300 mm × 4 m). The membranes were blocked for 60 min with TBS-T (0.1% Tween-20) supplemented by 5% BSA (Sigma-Aldrich, Saint Louis, MO, USA), and incubated overnight (4°C) with the following primary antibodies: anti-Phospho ERBB2 monoclonal antibody (1:500 dilution; #2243 Cell Signaling Technology, Inc., Danvers, MA, USA), anti-ERBB2 monoclonal antibody (1:1000 dilution; #4290 Cell Signaling Technology, Inc., Danvers, MA, USA), anti-Phospho ERBB3 (1:500 dilution; #4791 Cell Signaling Technology, Inc., Danvers, MA, USA), anti-ERBB3 (1:1000 dilution; #4754 Cell Signaling Technology, Inc., Danvers, MA, USA), anti-Phospho ERBB4 (1:500 dilution; #3790 Cell Signaling Technology, Inc., Danvers, MA, USA), anti-ERBB4 (1:500 dilution; #4795 Cell Signaling Technology, Inc., Danvers, MA, USA), and anti-GAPDH (1:1000 dilution; #G9545 Sigma-Aldrich, Saint Louis, MO, USA). For protein detection, the membrane was incubated with anti-rabbit horseradish peroxidase-labeled secondary antibody (Dako EnVision+ System- HRP Labelled Polymer) followed by a chemiluminescent reaction (Clarity Western ECL Substrate, Bio-Rad). Signals and images were acquired by Chemi Doc™ XRS 2015 (Bio-Rad Laboratories, Hercules, CA, USA), and densitometric analysis was performed using Image Lab software (version 5.2.1; Bio-Rad Laboratories, Hercules, CA, USA).

### Flow Cytometry Analysis of Cell Cycle Phases

700.000 SKBR3 cells were seeded on 6 cm plastic Petri dishes. After overnight starvation, cells were treated with neuregulin 1 (10 ng/mL), neuregulin 4 (10 ng/mL), trastuzumab (10 µg/mL) and pertuzumab (10 µg/mL), alone and in combination. After 48 hours of treatment cells were harvested, and fixed by slowly adding cold ethanol dropwise and then stored at -20°C overnight. The day after the samples were centrifuged, washed in PBS, and incubated with RNAse A and Propidium Iodide (PI) for 30 minutes at room temperature in the dark. After one wash in PBS, cells were resuspended in PBS and analyzed by CytoFLEX Flow cytometer through CytExpert software.

### Statistical Analysis

Statistical analyses were performed with GraphPad software (Prism 8). Whenever normality could be assumed the Student t-test 2-sided or analysis of variance (ANOVA) one-way and two-way, followed by Tukey’s or Sidak’s test was used to compare group means, as specified in the figure legends. P value < 0.05 was considered to represent a statistically significant difference. In all panels, numerical data are presented as mean + s.e.m.; results are marked with one asterisk (∗) if P <0.05, two (∗∗) if P <0.01, three (∗∗∗) if P <0.001 and four (∗∗∗∗) if P <0.0001.

## Data Availability Statement

The original contributions presented in the study are included in the article/**Supplementary Material**. Further inquiries can be directed to the corresponding author.

## Ethics Statement

All patients' data were derived from public databases, thus ethics approval was not required.

## Author Contributions

GD’U conceived the study. GD and CM designed the experiments. CM with the help of DR, MM, AM, SP, FS, CB, MS and EP performed the experiments and analyzed the data. GD’U and ML supervised the experiments done by their laboratory members, and GD’U supervised the entire project. CM and GD’U wrote the manuscript with editing contributions from all of the authors. All authors contributed to the article and approved the submitted version.

## Funding

This project was supported by AIRC to GDU (Grant number: MFAG 24684), by Fondazione Cariplo to GDU and ML (Grant Number: GR 2017-0800) and by Ministry of Health - Ricerca Corrente - IRCCS MultiMedica.

## Conflict of Interest

The authors declare that the research was conducted in the absence of any commercial or financial relationships that could be construed as a potential conflict of interest.

## Publisher’s Note

All claims expressed in this article are solely those of the authors and do not necessarily represent those of their affiliated organizations, or those of the publisher, the editors and the reviewers. Any product that may be evaluated in this article, or claim that may be made by its manufacturer, is not guaranteed or endorsed by the publisher.
