# Supplementary material for: Neuregulin 4 Boosts the Efficacy of Anti-ERBB2 Neutralizing Antibodies
Source: Front Oncol. 2022 May 18;12:831105. doi: 10.3389/fonc.2022.831105 (PMC9157648; doi:10.3389/fonc.2022.831105)
Supplement: Supplementary file 1 [file Image_1.pdf]

frontiers

Supplementary Material

1 Supplementary Figures

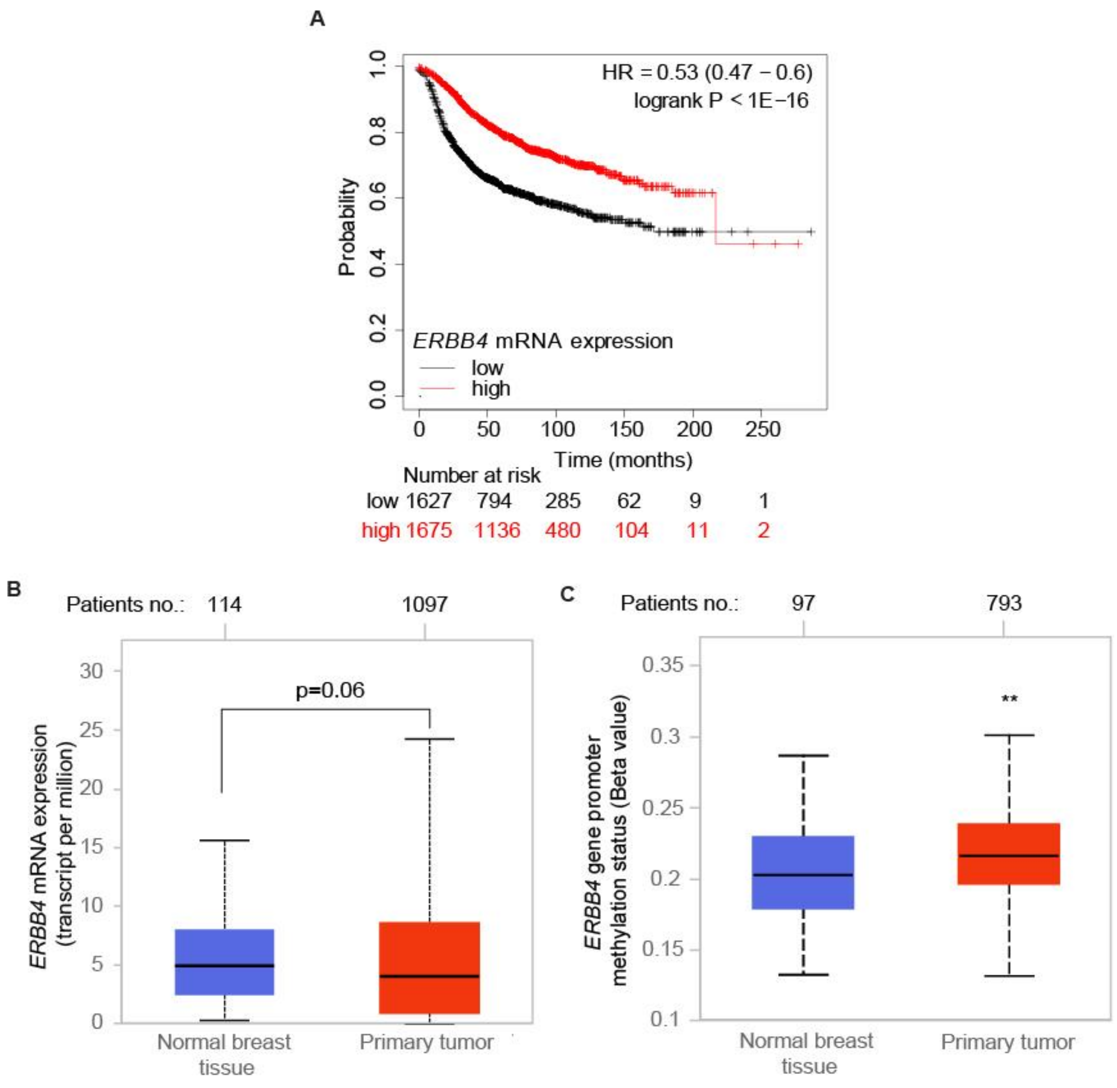

Supplementary Figure S1. *ERBB4* mRNA expression is reduced in breast cancer specimens compared to normal breast tissue and associates with longer-disease free patients' survival. (A) Relapse-free survival (RFS) of breast cancer patients stratified for *ERBB4* mRNA levels (trichotomization T1 vs T3) (n= 4929 patients); **(B)** *ERBB4* mRNA expression levels in normal breast tissue compared to primary breast tumors (n = 1211 patients); **(C)** Levels of DNA methylation of ERBB4 promoter in normal breast tissue compared to primary breast tumors (n = 890 patients). ** $p < 0.01$.

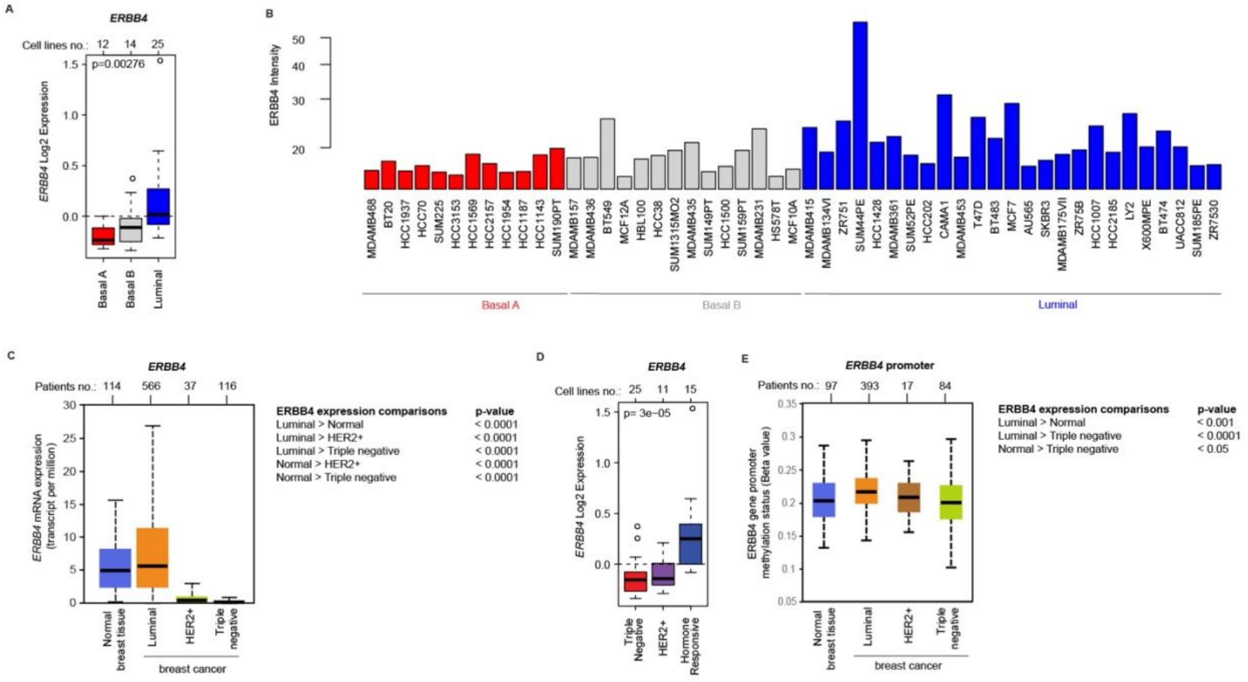

Supplementary Figure S2. *ERBB4* mRNA expression and promoter methylation analysis in normal tissue, breast cancer patients or in breast cancer cell lines stratified for clinical and molecular subtypes. (**A-B**) *ERBB4* mRNA expression in breast cancer cell lines (n = 51 cell lines) stratified for molecular clusters (average in **A**, individual values in **B**); (**C**) *ERBB4* mRNA expression levels in breast normal tissue and breast cancer patients stratified for clinical subtypes (n = 1843 patients); **(D)** *ERBB4* mRNA expression in breast cancer cell lines stratified for clinical subtypes (triple-negative, HER2+ and hormone-responsive) (n = 51 cell lines); (**E**) Levels of DNA methylation of ERBB4 promoter in breast normal tissue and breast cancer patients stratified for clinical subtypes (n = 591 patients).

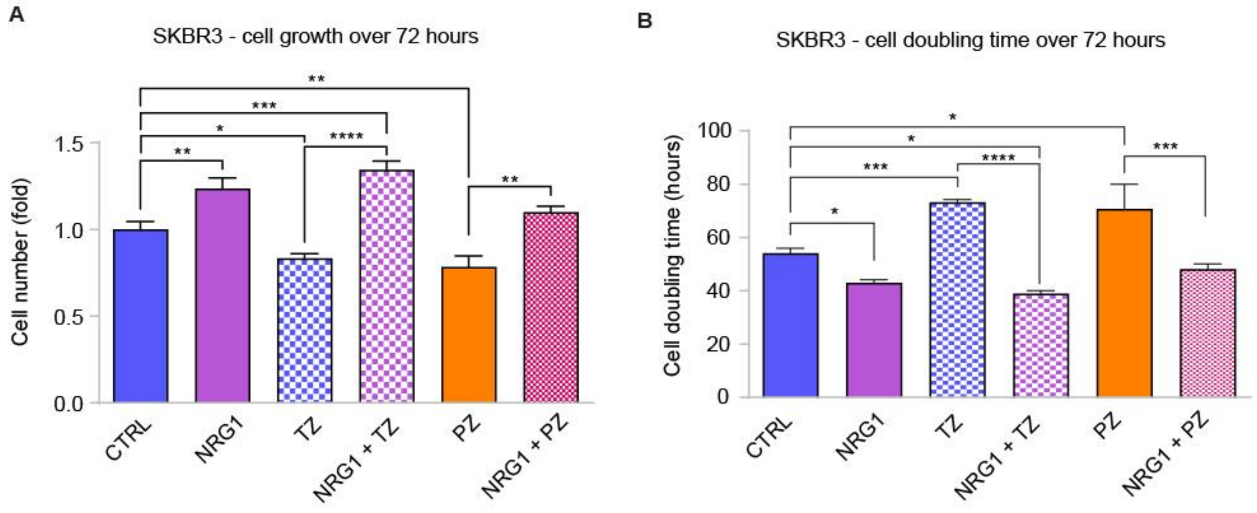

Supplementary Figure S3. Analysis of SKBR3 cell proliferation after treatment with NRG1 and anti-HER2 agents (trastuzumab and pertuzumab). (**A-B**) Cell proliferation analysis by Livecyte technology of SKBR3 cells treated with/without NRG1 (10 ng/mL), alone or in combination with trastuzumab (10 µg/mL) or pertuzumab (10 µg/mL). Cell number and cell doubling time at 72 hours are provided in **A** and **B** respectively. In panel **A** numerical data are normalized to control cells. In all panels, numerical data are presented as mean (error bars show s.e.m.); statistical significance was determined using one-way ANOVA followed by Tukey's test; * $p < 0.05$, ** $p < 0.01$, *** $p < 0.001$ and **** $p < 0.0001$.

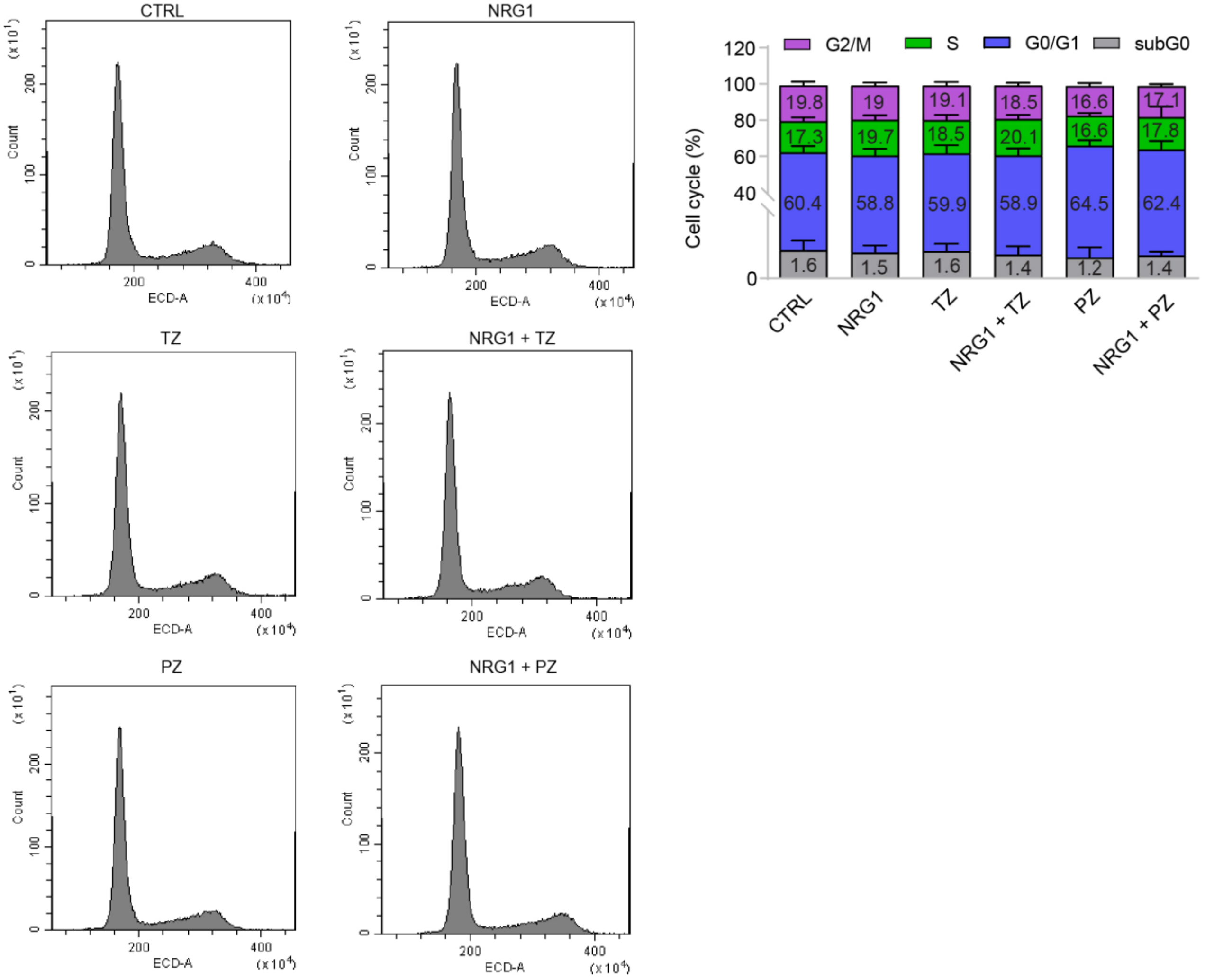

Supplementary Figure S4. Cell cycle analysis upon combinatorial administration of NRG1 and anti-HER2 agents. Cell cycle analysis of SKBR3 cells treated with NRG1 (10 ng/mL), alone or in combination with trastuzumab (10 µg/mL) or pertuzumab (10 µg/mL) for 48 hours. Representative images of DNA content distribution of cells stained with Propidium Iodide and analyzed by flow cytometry. Histograms show the percentage of cells in the different phases of the cell cycle. Data were processed through CytExpert software.

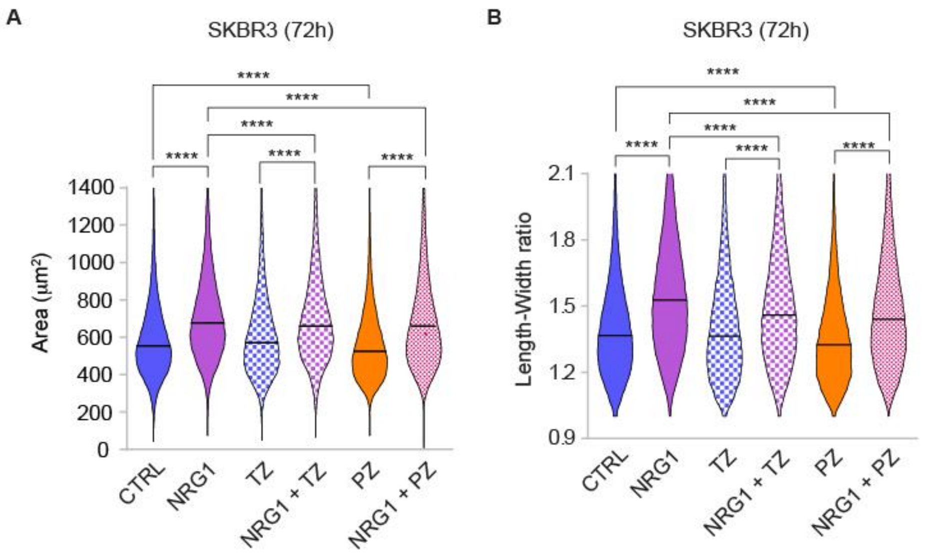

Supplementary Figure S5. Analysis of morphological parameters of SKBR3 cells after treatment with NRG1 and anti-HER2 agents (trastuzumab and pertuzumab). (A-B) Analysis of morphological parameters, namely (**A**) cells area and (**B**) length-to-width ratio in SKBR3 cells cultured *in vitro* and treated with/without NRG1 (10 ng/mL), alone or in combination with trastuzumab (10 µg/mL) or pertuzumab (10 µg/mL), for 72 hours. In all panels, numerical data are presented as mean (error bars show s.e.m.); statistical significance was determined using one-way ANOVA followed by Tukey's test; **** $p < 0.0001$.

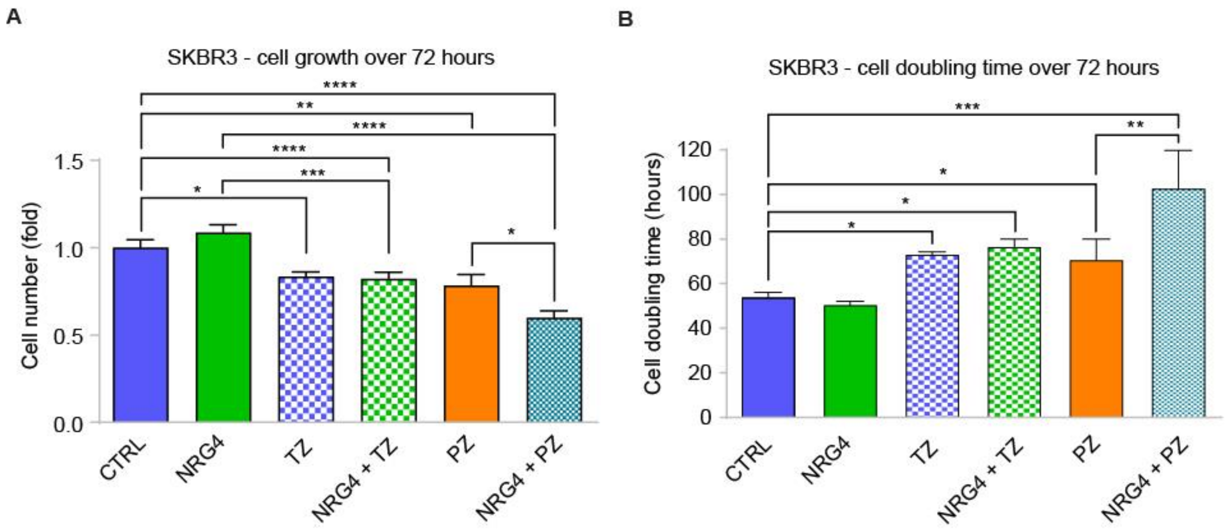

Supplementary Figure S6. Analysis of SKBR3 cell proliferation after treatment with NRG4 and anti-HER2 agents (trastuzumab and pertuzumab). (A-B) Cell proliferation analysis by Livecyte technology of SKBR3 cells treated with/without NRG1 (10 ng/mL), alone or in combination with trastuzumab (10 µg/mL) or pertuzumab (10 µg/mL). Cell number and cell doubling time at 72 hours are provided in **A** and **B** respectively. In panel **A** numerical data are normalized to control cells. In all panels, numerical data are presented as mean (error bars show s.e.m.); statistical significance was determined using one-way ANOVA followed by Tukey's test; * $p < 0.05$, ** $p < 0.01$, *** $p < 0.001$ and **** $p < 0.0001$.

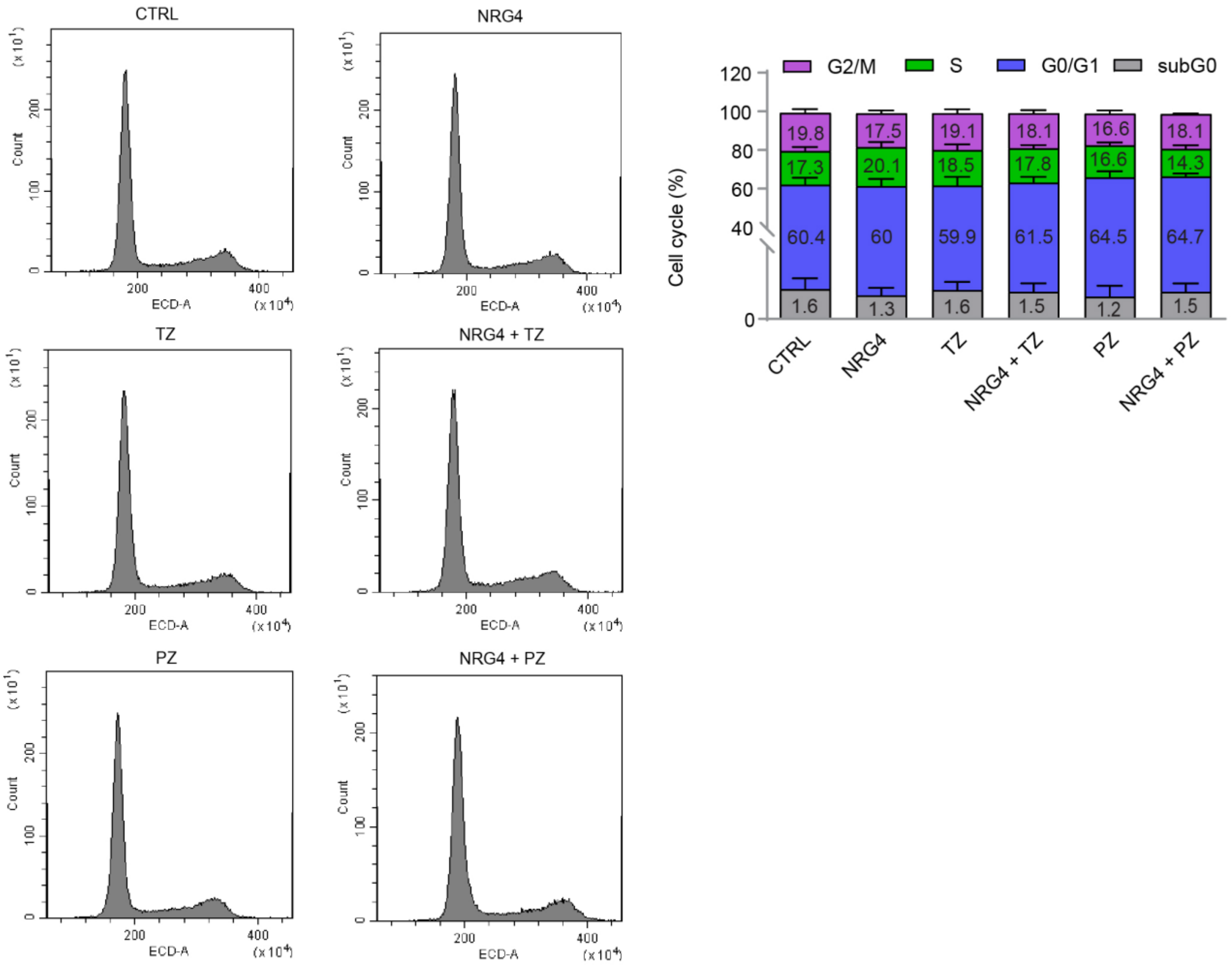

Supplementary Figure S7. Cell cycle analysis upon combinatorial administration of NRG4 and anti-HER2 agents. Cell cycle analysis of SKBR3 cells treated with NRG4 (10 ng/mL), alone or in combination with trastuzumab (10 µg/mL) or pertuzumab (10 µg/mL), for 48 hours. Representative images of DNA content distribution of cells stained with Propidium Iodide, and analyzed by flow cytometry. Histograms show the percentage of cells in the different phases of the cell cycle. Data were processed through CytExpert software.

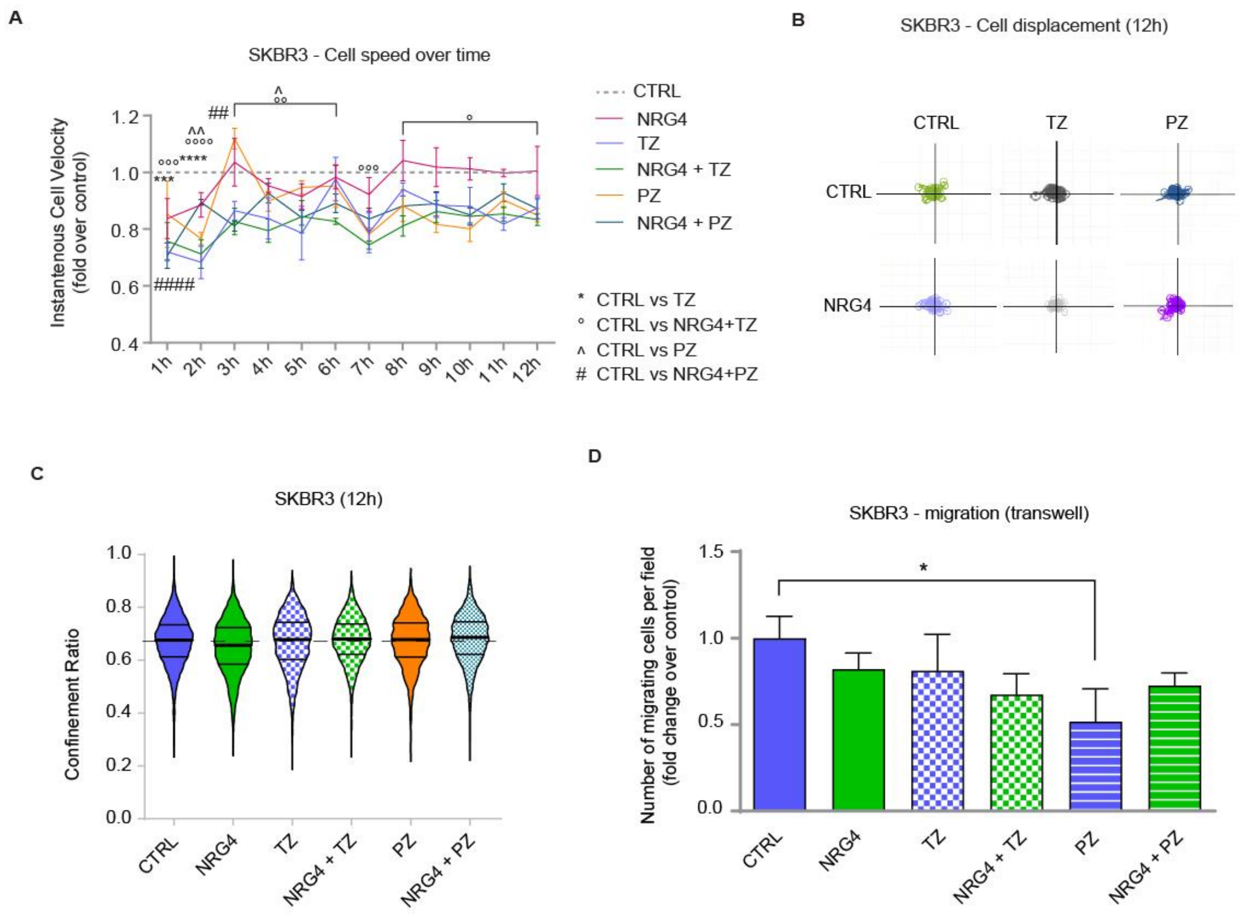

Supplementary Figure S8. Analysis of SKBR3 cell motility and migration after treatment with NRG4 and anti-HER2 agents (trastuzumab and pertuzumab). (A) Cell speed analysis over time by Livecyte technology of SKBR3 cells treated with/without NRG4 (10 ng/mL), alone or in combination with trastuzumab (10 µg/mL) or pertuzumab (10 µg/mL). Cell velocity has been detected every hour up to 12 hours; and normalized to control cells (dotted line); (**B**) SKBR3 cell displacement analysis by Livecyte technology. Representative track plots of SKBR3 treated with/without NRG4 (10 ng/mL), alone or in combination with trastuzumab (10 µg/mL) or pertuzumab (10 µg/mL) up to 12 hours are provided; (**C**) Confinement ratio analysis of SKBR3 cells by Livecyte technology. Violin plots of SKBR3 treated with/without NRG4 (10 ng/mL), alone or in combination with trastuzumab (10 µg/mL) or pertuzumab (10 µg/mL) for 12 hours; **(D)** Transwell migration assay of SKBR3 cells treated with/without NRG4 (10 ng/mL), alone or in combination with trastuzumab (10 µg/mL) or pertuzumab (10 µg/mL) for 24 hours. In all panels, numerical data are presented as mean (error bars show s.e.m.); statistical significance was determined using two-way ANOVA in **A** and one-way ANOVA in **C**, **D** followed by Tukey's test; 1 symbol (*, ^, °) $p < 0.05$, 2 symbols (^^, °°) $p < 0.01$, 3 symbols (***,°°°) $p < 0.001$, 4 symbols (****, °°°°) $p < 0.0001$.

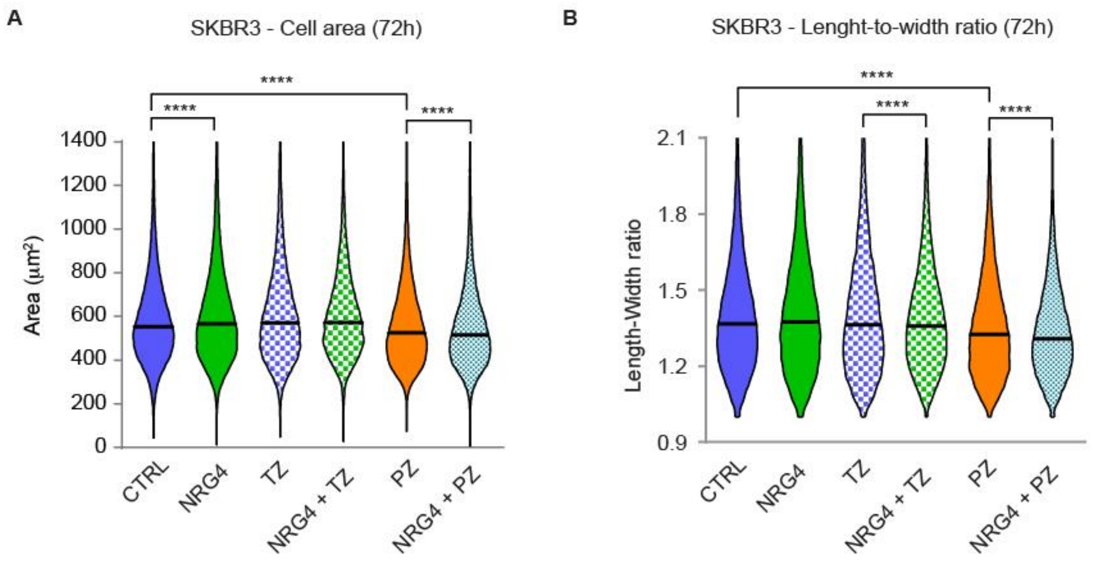

Supplementary Figure S9. Analysis of morphological parameters of SKBR3 cells after treatment with NRG4 and anti-HER2 agents (trastuzumab and pertuzumab). (A-B) Analysis of morphological parameters, namely (**A**) cells area and (**B**) length-to-width ratio in SKBR3 cells cultured *in vitro* and treated with/without NRG4 (10 ng/mL), alone or in combination with trastuzumab (10 µg/mL) or pertuzumab (10 µg/mL), for 72 hours. In all panels, numerical data are presented as mean (error bars show s.e.m.); statistical significance was determined using one-way ANOVA followed by Tukey's test; **** $p < 0.0001$.
